# Kennedy class III and IV dental arches: Trueness analysis of digitization methods and 3D-printing step

**DOI:** 10.1590/0103-6440202405980

**Published:** 2024-12-06

**Authors:** Lucas Moreira Mendonça, Marianna Soares Nogueira Borges, Ayodele Alves Amorim, Bruna Neves de Freitas, Camila Tirapelli

**Affiliations:** 1 Department of Dental Materials and Prosthodontics, School of Dentistry of Ribeirão Preto, University of São Paulo, Ribeirão Preto, SP, Brazil; 2 Department of Dentistry and Oral Health, Aarhus University, Aarhus, Denmark

**Keywords:** dental arch, 3d printing, dental impression technique, edentulous, intraoral scanner

## Abstract

This study aims to evaluate the trueness of Kennedy Class III and IV dental arches digitized by different methods and three-dimensionally (3D) printed using stereolithography technology in an in vitro setup. Reference casts (maxillary Kennedy class III and IV) were produced by computer assisted design and manufacture, and linearly measured at occlusocervical, interarch, and edentulous space dimensions. Intraoral scanner (IOS), extraoral scanner (EOS) and cone beam computed tomography (CBCT) digitized the reference casts. Each digital file was 3D-printed using stereolithography technology, totalizing sixty experimental casts (n=10 per group). The same measurements taken from the reference casts were performed on experimental casts. Two-way ANOVA and Bonferroni post-test were used for trueness (distortion between the experimental and reference casts). Distortion was significantly greater for class IV when compared with class III and increased after the 3D-printing step. Among digitizing methods, IOS and EOS had a similar performance and casts from CBCT showed higher distortion, reaching -1.0 and -1.4 mm in the edentulous spaces of digital and 3D-printed cast, respectively. It was possible to conclude that the trueness of Kennedy class III and IV arches were different according to digitizing processes with higher distortion at the edentulous spaces when the cast was digitized by CBCT and converted to a 3D model, compared to IOS and EOS; and in the Kennedy class IV dental arch condition.

## Resumo

Este estudo tem como objetivo avaliar a veracidade de arcadas dentárias Classe III e IV de Kennedy digitalizadas por diferentes métodos e impressas tridimensionalmente (3D) usando tecnologia de estereolitografia em cenário in vitro. Modelos de referência (maxilares classes Kennedy III e IV) foram produzidos por desenho e manufatura assistidos por computador e medidos linearmente nas dimensões oclusocervicais, interarcos, e no espaço edêntulo. Scanner intraoral (IOS), scanner extraoral (EOS) e tomografia computadorizada de feixe cônico (CBCT) digitalizaram os modelos de referência. Cada arquivo digital foi impresso em 3D utilizando tecnologia de estereolitografia, totalizando sessenta moldes experimentais (n=10 por grupo). As mesmas medidas retiradas dos modelos de referência foram realizadas nos modelos experimentais. Two-way ANOVA e pós-teste de Bonferroni foram utilizados para veracidade (distorção entre os modelos experimental e de referência). A distorção foi significativamente maior para a classe IV quando comparada com a classe III e aumentou após a etapa de impressão 3D. Entre os métodos de digitalização, IOS e EOS tiveram desempenho semelhante e os modelos da CBCT apresentaram maior distorção, atingindo -1.0 e -1.4 mm nos espaços edêntulos de modelos digitais e impressos em 3D, respectivamente. Foi possível concluir que a veracidade dos arcos classes III e IV de Kennedy foram diferentes de acordo com os processos de digitalização com maior distorção nos espaços edêntulos, quando o modelo foi digitalizado por CBCT e convertido para um modelo 3D em comparação com IOS e EOS; e na condição de arcada dentária classe IV de Kennedy.

## Introduction

New technologies in Dentistry, such as computer-aided imaging, design, and manufacturing, have introduced options to workflows within dental offices and prosthetic laboratories ^1^ ^,^ ^2^. However, the digitization of the patient’s dental arch plays an important role and is shown to be affected by numerous factors related to the patient and operator, impacting the trueness of the entire workflow from the outset ^2^ ^,^ ^3^. Scientific evidence suggests that the trueness of intraoral scanning decreases as the edentulous space increases and may depend on its length and location within the arch ^4^ ^,^ ^5^. Studies have also reported that different digitization methods can yield varying trueness values due to differences in imaging acquisition technology ^6^ ^,^ ^7^. Particularly in cases of edentulous spaces involving the midline segment and posterior limited by teeth, the intraoral scanning poses challenges due to arch curve and distance between the scanner head and the surface ^8^ ^,^ ^9^. Nevertheless, investigations encompassing these scenarios, such as those involving the digital workflow on Kennedy class III and IV, are scarce ^5^, and currently, there is no available research considering the setup of testing it with three different digitization methods and including the 3D-printing step.

One of the digitizing methods available is desktop optical scanner, an extraoral scanner (EOS) widely used in dental laboratories, which allows dental stone casts to be digitized and integrated into the digital workflow ^7^. Additionally, there are other technologies for digitizing patients’ intraoral structures such as intraoral optical scanner (IOS) and cone beam computed tomography (CBCT) ^1^ ^,^ ^2^. IOS has been shown to be affected by the absence of teeth, due to the limited field of view of the scanner head and lack of reference points, requiring extensive stitching during scanning, in contrast with desktop scanners with a broader scan area ^3^ ^,^ ^10^. In turn, CBCT has been explored due to its application to create full-digitalized patients, and potential use as an alternative when optical scanners are unavailable or impractical to use (e.g., limited mouth opening and areas beyond the scanner light range) and the patient has previous records ^6^. There is evidence in literature regarding the influence of digitizing and 3D-printing methods on the trueness of dental arch cast. De Freitas et al. ^11^ compared the trueness of digital and 3D-printed casts with different digitizing (CBCT and IOS) and 3D-printing methods. It was found that the trueness of digital and 3D-printed casts was influenced by digitizing and printing methods ^11^. However, the results from this study came from a complete dentate arch. In this context, in 2021, Carneiro Pereira et al. ^12^ ^)^ evaluated whether computer assisted design and manufacture would be accurate for the framework manufacturing for removable partial dentures. In total, seven studies were reviewed; among them, one study compared EOS with IOS and found comparable results for trueness ^12^. However, only class I and III were investigated. Kontis et al. ^13^ compared the accuracy of two different IOS on different mandibular teeth gaps (Kennedy classes III, III modification 1, IV and complete dentate arch) and found differences between IOS and the different edentulous arches conditions. In this sense, it would be interesting to evaluate digitizing methodologies for different classifications of partially edentulous maxilla, including class IV that is poorly studied and represent the greater challenge in terms of edentulous region to be digitized, and the 3D-printing step.

Therefore, the objective of this in-vitro study is to compare the trueness of Kennedy class III and IV dental arches digitized with different technologies (IOS, EOS, CBCT) and 3D-printed using stereolithography technology. The null hypothesis was that there is no difference in trueness of digital and 3D-printed casts considering different edentulous arch conditions and digitizing methods.

## Materials and Methods

### Study design

**Figure 1:**
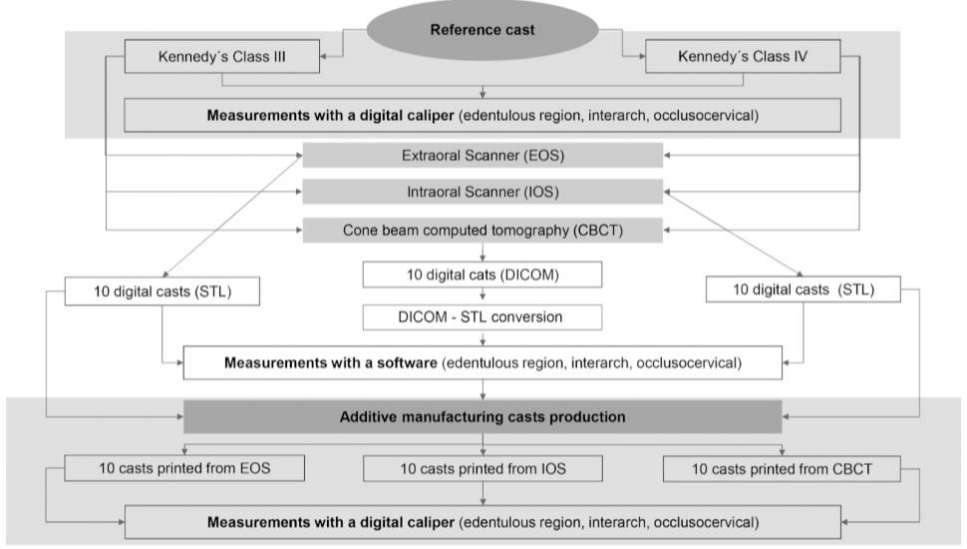
Flowchart of study design. IOS: intraoral scanning. EOS: Extraoral scanning. CBCT: cone beam computed tomography.

This is an in-vitro study in which trueness was the variable analyzed through distortion (difference between experimental and reference casts). The factors under variation were different edentulous spaces (Kennedy class III and IV) and digitizing methods (EOS, IOS and CBCT), as shown in Figure 1. The sample size was defined using the G*Power 3.1.9.6 software (Heinrich-Heine-Universität Düsseldorf, Düsseldorf, Germany) considered a test with 80% of power and 95% of confidence. The 60 digital casts and the 60 solid casts were distributed among the experimental groups allocating 10 casts per experimental group.

### Reference casts

**Figure 2:**
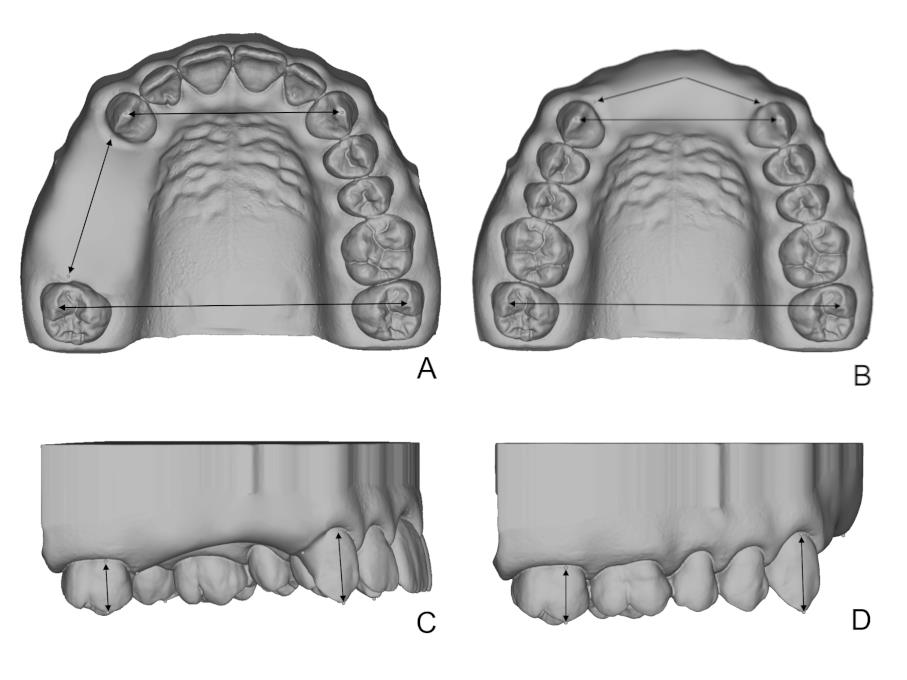
Visual demonstration of dental arch casts and dimensions taken from reference cast and experimental groups: a and b) Occlusal view of Kennedy class III and IV dental arches, respectively, showing interarch (distance between tip of the canines cusps and between the tip of the mesiobuccal cusps of second molars) and edentulous space dimensions (distance between reference points located at the mesiodistal boundaries of the edentulous spaces); c and d) Lateral view of Kennedy class III and IV dental arches, respectively, showing occlusocervical dimensions (distance between reference points located at cervical third of the buccal surface of the canines and second molars to their respective cusps tips). Note the reference measurement points at the tips of each arrow.

A maxillary complete dentate typodont was digitized by a desktop scanner (UP300, Up3d, Shenzhen, Guangdong, China). In the Standard Tessellation Language (STL) file, edentulous spaces and reference measurement points (Figure 1) were created (Meshmixer version 3.5, Autodesk Inc, San Rafael, California, United States), allowing that the only difference between the reference casts were in the edentulous spaces. The reference measurements points were digitally designed based on Jaber et al. ^14^, consisting of ball-shaped markers 0.5 mm diameter. They were located considering interarch, occlusalcervical, and edentulous space dimensions, as follows: at the tip of canines cusps, the tip of the mesiobuccal cusps of second molars, the cervical third of the buccal surface of the canines and second molars in the direction of the aforementioned cups, and mesiodistal boundaries of the edentulous spaces (Figure 2). The teeth of these reference points were defined for being adjacent to both edentulous conditions addressed in this study. The reference casts were produced by additive manufacturing with the following 3D-printing procedures: STL files were processed to remove excess mesh, make solid, hollow, and set thickness (2 mm) for 3D-printing. Then, the files were sent to 3D-printer software (Photon Workshop v2 slicer, Anycubic Technology Co., Shenzhen, Guangdong, China) in which the following characteristics were determined: 90 degrees to the horizontal plane, supports addition using “automatic support” tool, thickness of 0.05 mm for each layer, time exposure of 10 seconds for each layer, and time exposure of base layer of 60 seconds, being the composed of 3 layers. 3D-printed casts were manufactured by a stereolithography printer (Photon Zero printer, Anycubic Technology Co., Shenzhen, Guangdong, China). An acrylate photopolymer resin material (stOne model; dOne 3D, Ribeirão Preto, São Paulo, Brazil) was used with the following characteristics: beige color, Shore D hardness of 82, density of 1.1 g/mm³, viscosity of 700-1200 cps and flash point >95 ºC, in addition to being sensitive to 405 nm light. Reference casts were produced simultaneously using about 18 ml of acrylic resin and a time of 5 hours and 20 minutes. After 3D-printing, the casts were washed using isopropyl alcohol and post-cured with UV Led (405 nm) for 10 minutes in a specialized machine (Anycubic Wash and Cure 2.0, Anycubic Technology Co., Shenzhen, Guangdong, China). Thus, the casts were stored indoors in plastic boxes, at room condition (25-30 ºC), out of light, and measured the day after (24 hours). The measurements were taken three times by the same operator using a 150 mm digital caliper (Mitutoyo, Kawasaki, Kanagawa, Japan) presenting an accuracy of ±0.02 according to the manufacturer, considering the distances between the reference points located at: occlusocervical length of the teeth adjacent to edentulous spaces, interarch, and edentulous space for both Kennedy’s class conditions, as illustrated in Figure 2. The average of these 3 measurements were considered as the measure obtained at each dimension.

### Digital and 3D-printed casts

Each reference cast was digitized ten times by an experienced operator using the IOS device (CEREC Omnicam, Dentsply Sirona, Charlotte, North Carolina, United States) which employs a combined optical triangulation and confocal microscopy-based technology. The scanning technique followed the manufacturer’s instructions. STL data files (n=10) were obtained for each reference.

On the EOS device (UP300, Up3d), the reference cast was positioned and digitized using the model scan mode for 20 seconds. The scanner employs non-contact customized blue light technology and two 1.3 MP cameras. This step was performed for both class III and IV reference casts. At the end, ten STL data files (n=10) were obtained for each reference cast.

On the CBCT equipment (Eagle 3D, Dabi Atlante, Ribeirão Preto, São Paulo, Brazil), reference casts were digitized ten times each using with the following image acquisition parameters: voxel of 0.10 mm, 25.5 seconds, 85 kV, 4 mA and a field of view of 6x8 cm. DICOM files were obtained for each reference cast (n=10). DICOM dataset was converted to STL file by an experienced operator through segmentation process (InVesalius 3.1 software, Renato Archer Information Technology Center, Campinas, São Paulo, Brazil) required to conversion to STL file, using a manual threshold value of -170 to 7135 shades of gray determined into software ^16^.

3D-printed casts were randomly manufactured (ten per group), totalizing one hundred and twenty experimental casts (sixty digital and sixty 3D-printed). The same 3D-print setup as aforementioned was used for the experimental casts. Two casts were printed at each time, totaling 5 days of experimental casts production.

### Measurements

Based on the literature ^15^, this study performed linear measurements on the experimental casts the day after their production. The same measurements taken from the reference cast were also taken from digital casts using a digital ruler tool (Orthoview software, 3Shape, Copenhagen, Denmark) and from the 3D-printed cast using the aforementioned digital caliper and by the same operator (Figure 2). Measurements were done three times considering the distance between the reference points shown on Figure 2, and the average of these 3 measurements was considered as the measure obtained at each dimension. The linear measurements were performed on all the casts in the following sequence: occlusocervical, interarch and edentulous space.

### Data analysis

Trueness was evaluated as distortion (experimental minus reference cast). Two-way ANOVA and Bonferroni post-test were used to provide a pairwise comparison for digital casts and 3D-printed casts from the same and different Kennedy’s class. All analyses were done for each dimension separately. In all tests, the level of significance was set at P≤.05 and calculations were performed using statistical software (GraphPad Prism, version 9.4.1 for Windows, GraphPad Software, Boston, Massachusetts, United States).

## Results

**Figure 3:**
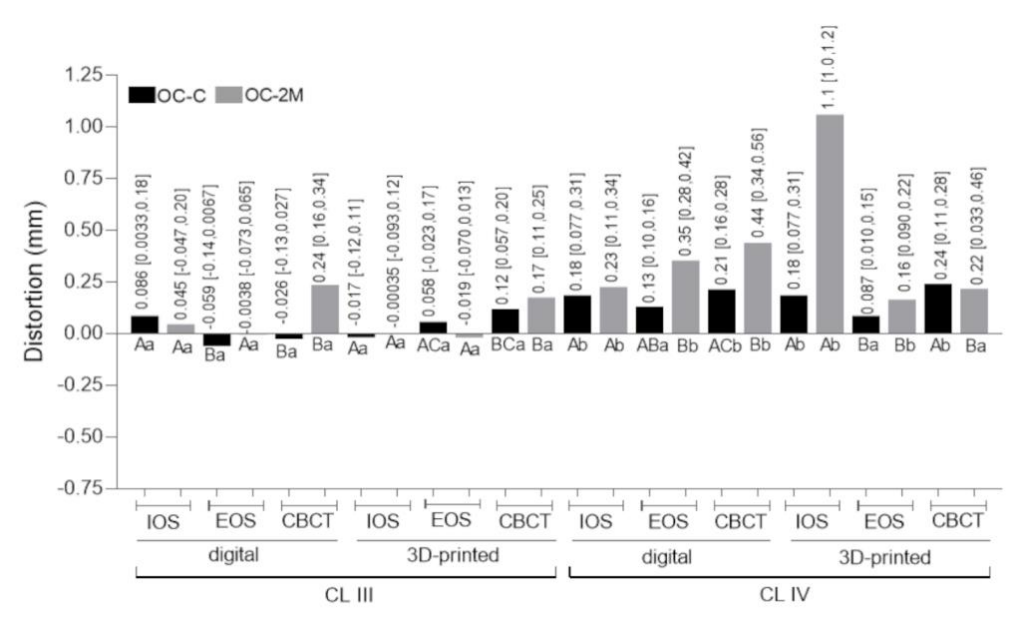
Trueness considering the distortion from occlusocervical (OC) dimensions in Kennedy class III and IV. OC-C: occlusocervical dimension from canine; OC-2M: occlusocervical dimension from second molar. Numerical values indicate the distortion (mm) and confidence interval. The same color bars are compared: different capital letters indicate statistical difference between casts (digital vs digital and 3D-printed vs 3D-printed) for the same arch classification; different lower-case letters indicate difference between casts (digital vs digital and 3D-printed vs 3D-printed) for different arch classifications.

**Figure 4:**
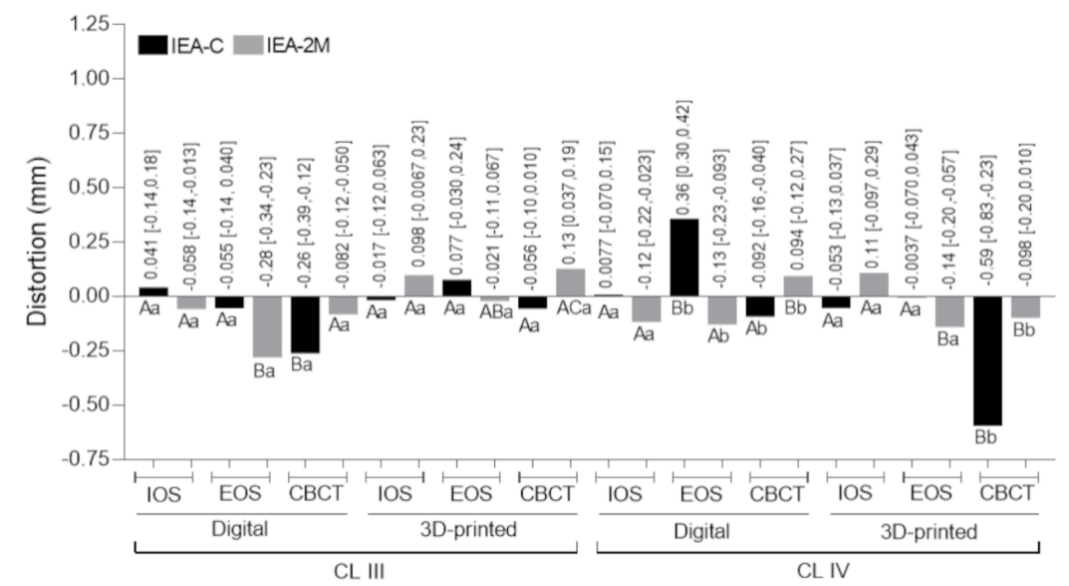
Trueness considering the distortion from interarch (IEA) dimensions in Kennedy class III and IV. IEA-C: interarch dimension between canines; IEA-2M: interarch dimension between second molars. Numerical values indicate the distortion (mm) and confidence interval. The same color bars are compared: different capital letters indicate statistical difference between casts (digital vs digital and 3D-printed vs 3D-printed) for the same arch classification; different lower-case letters indicate difference between casts (digital vs digital and 3D-printed vs 3D-printed) for different arch classifications.

**Figure 5:**
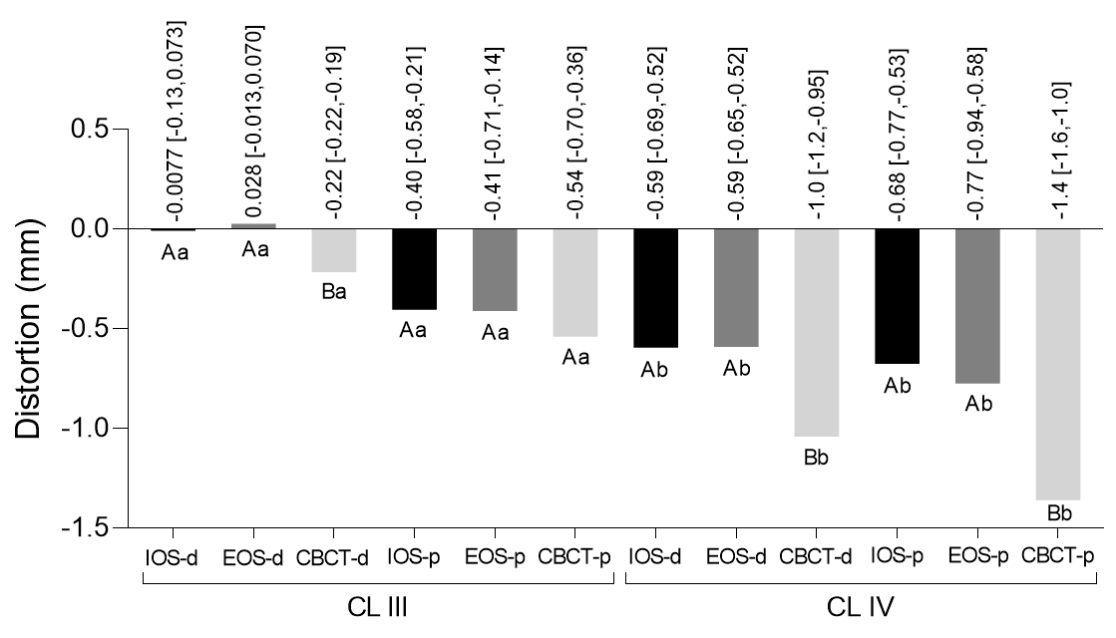
Trueness considering the distortion from edentulous space in the Kennedy class III and IV. Numerical values indicate the distortion (mm) and confidence interval. The same color bars were compared: different capital letters indicate statistical difference between casts (digital vs digital and 3D-printed vs 3D-printed) for the same arch classification; different lower-case letters indicate difference between casts (digital vs digital and 3D-printed vs 3D-printed) for different arch classifications

Figures 3, 4 and 5 show the distortion among groups considering the occlusocervical, interarch, and edentulous space dimensions, respectively.

For class IV, at occlusocervical and interarch dimensions, highest values of distortion were shown in 3D-printed casts, such as the 1.1 mm at molar occlusocervical (from IOS) and -0.59 mm at molar interarch measurements (from CBCT), as shown in Figure 3 and 4. Considering edentulous space, distortion at class IV showed most of values above the 0.5mm and it was possible to observe significant differences between digitizing methods for digital and 3D-printed casts from CBCT showing -1.0 mm and -1.4 mm, respectively (Figure 5).

Greater and statistically significant different distortions were found for Kennedy class IV when comparing its edentulous space to that of class III, in both digital and 3D-printed casts, as shown in Figure 5.

## Discussion

This study compared the trueness of digital and 3D-printed dental arch casts with two different partially edentulous arches, Kennedy class III and IV. The findings revealed that trueness varied significantly depending on the edentulous condition and digitizing method. As a result, the study rejected the null hypotheses as trueness was different among digital and 3D-printed casts considering different edentulous arch conditions and digitizing methods.

Discussing the results found, the significant differences that appeared when comparing digital casts can be attributed to the influence of the digitizing method, dental arch condition, and DICOM conversion process ^2^ ^,^ ^4^ ^,^ ^6^. In this study, CBCT showed the worst trueness whereas IOS and EOS showed similar performance. This finding may result from the conversion process required to obtain a 3D model from CBCT images. This process requires segmentation of patients’ structures, and its accuracy is operator- and method- dependent, involving interpretation of structures boundaries and partial volume effect in voxels located at the edges of the object ^6^ ^,^ ^16^. In this context, segmentation methods have been developed aiming to reduce operator influence, take account of the technical aspects involved in a CBCT data, and increase accuracy ^16^, potentially reducing the differences in trueness compared to the other scanners found in the present study. Another important aspect to be considered when obtaining 3D models from CBCT data is to consider the voxel size of CBCT equipment, as it can limit its accuracy and impact the volume segmentation ^16^ ^,^ ^17^. Considering the voxel size of the CBCT equipment used in this study and previous literature ^6^ ^,^ ^17^, the primary influence on distortion results would come from the segmentation process as described above. It is worth mentioning that this study included CBCT since it represents a possibility of obtaining a patient's 3D model if previous scans were taken, mainly in case of patient-related limitations to intraoral scanner or conventional impression (e.g. limited mouth opening, and areas beyond the scanning light range). As CBCT uses ionizing radiation, its choice must comply with ALADA and ALADAIP principles ^18^, while also recognizing its limitations in reproducing surfaces ^19^, especially if high-density involved are present causing imaging artifacts ^20^. In this regard, and considering the results found, optical scanners should be preferred wherever possible for obtaining the 3D model of patients’ intraoral structures.

Comparing the results of digital casts with literature and variables in common in this investigation, Hayama et al. ^10^ compared digital casts with Kennedy class I and III classifications, digitized with an IOS and evaluated meshes using superimposition technique. The authors observed that IOS showed better trueness compared to conventional impressions ^10^. Another study ^21^ also compared different IOS within three groups, varying age and arch (complete or partial) and concluded that the devices had limitations in the accuracy of full-arch impressions and that fully dentition or partially edentulous dentitions with small gaps did not influence the outcome ^21^. Kontis et al. ^13^ evaluated accuracy in mandibular casts with different Kennedy classes (III, III modification 1, IV and complete dentate arch) and digitized it with two different IOS. The results showed that Primescan performed better than Omnicam and that class III resulted in low accuracy by Primescan; while Omnicam produced the lowest accuracy in most parameters for class III cast modification 1 ^13^. The authors concluded that both IOS investigated were sufficiently accurate for this purpose; however, IOS technology and the presence of edentulous space influenced the accuracy of the digital cast ^13^. Ellakany et al. ^7^ compared a full-arch impression taken by IOS and EOS and found that while both scanning modalities had similar accuracy for measurements of premolar and molars, while EOS performed better in canines ^7^. Although IOS and EOS also demonstrated similar performance in many measurements in the present study, it should be noted that this may be attributable to the in-vitro setting and a well-trained operator. EOS has been considered a more predictable digitizing method because it operates on dental models, without interference from saliva, being unaffected by variations in inclination and light conditions imposed and restricted by soft tissues in the oral environment ^2^ ^,^ ^22^ ^,^ ^23^. Additionally, it requires less stitching due to its wider scan area compared to IOS, which must be limited as an intraoral device ^24^.

From the comparison of 3D-printed casts, it is highlighted the values of distortion that exceeded 0.5mm ^6^ ^,^ ^25^, as the distortion of 1.1 mm at occlusocervical measurement in molar and the distortion at edentulous space in both classes III and IV (class IV shown a distortion of -1.0 mm when digitized with CBCT and -1.45 mm after 3D-printing). In this context, it is important to discuss the multiple interfering factors that can be present in the 3D-printing process. In this study, the casts were printed at 90 degrees angle to the base of the impression that supported on the distal region of the second molar, which means that this region may have experienced a pull-back of the support during the printing, justifying this deviating value only in the region of molar. In addition, the posterior region would be more susceptible to distortions starting from the digitization by the intraoral scanning due to the shape of dental arch broadening buccally from anterior to the posterior segment. ^26^. A point that can justify the increased distortion at edentulous space of class IV was its extension and location. In this study class IV is more extensive than class III, a factor that also causes more distortion due to the lack of reference during digitization. Besides, the curved shape of class IV may have influenced the digitizing and printing process, because it can suffer great shrinkage during the polymerization phase of impression material ^4^ ^,^ ^27^.

The presented results must be considered taking into account two main aspects: one concerning the measurements method and the other pertaining to the in-vitro setting. While linear measurements are commonly employed in dental arch examinations (e.g., for orthodontic purposes), this method is unable to detect distortions throughout the entire arch. In this study, the term “distortions” refers to each linear measurement taken and compared directly to the initial object (reference cast). Therefore, it should not be interpreted as a distortion of the entire arch. To visualize distortions throughout the arch, the superimposition comparison method using metrology software would be indicated; nevertheless, this method also requires careful consideration of the software used and the alignment technique applied ^28^. The second aspect concerns the inherent limitations of an in-vitro setting. The absence of clinical factors such as soft tissues, patient movement, saliva, natural color and reflectance of oral structures may influence the conditions under which the impressions would be taken and their respective performance in a clinical scenario ^3^ ^,^ ^23^. For future investigations, studies comparing other edentulous spaces and clinical studies comparing different imaging and manufacturing technologies are necessary to determine the optimal digitizing method for edentulous conditions.

## Conclusion

In conclusion, the trueness of digital and 3D-printed casts was influenced by different edentulous conditions and digitizing methods, and it varied depending on the location of the measurement. Casts digitized using CBCT followed by a conversion process to a 3D model showed greater distortion in the edentulous space compared to intraoral and desktop scanners in both digital and 3D-printed modality. The 3D-printed casts also exhibited more distortions than the digital ones in the edentulous spaces. Kennedy class IV presented higher distortion compared to class III in most dimensions.
